# Electrical Conduction Properties of Hydrogenated Amorphous Carbon Films with Different Structures

**DOI:** 10.3390/ma14092355

**Published:** 2021-05-01

**Authors:** Masashi Tomidokoro, Sarayut Tunmee, Ukit Rittihong, Chanan Euaruksakul, Ratchadaporn Supruangnet, Hideki Nakajima, Yuki Hirata, Naoto Ohtake, Hiroki Akasaka

**Affiliations:** 1 Department of Mechanical Engineering, Tokyo Institute of Technology, 2-12-1, O-okayama, Meguro-ku, Tokyo 152-8550, Japan; masa.tmdkr@gmail.com; 2 Synchrotron Light Research Institute (Public Organization), 111 University Avenue, Nakhon Ratchasima 30000, Thailand; sarayut@slri.or.th (S.T.); ukit@slri.or.th (U.R.); chanan@slri.or.th (C.E.); ratchadaporn@slri.or.th (R.S.); hideki@slri.or.th (H.N.); 3 Institute of Innovative Research, Tokyo Institute of Technology, 4259 Nagatsuta, Midori-ku, Yokohama, Kanagawa 226-8503, Japan; hirata.y.ac@m.titech.ac.jp (Y.H.); ohtake.n.aa@m.titech.ac.jp (N.O.)

**Keywords:** hydrogenated amorphous carbon film, electrical conduction, CVD deposition, near-edge X-ray absorption fine structure, variable range hopping

## Abstract

Hydrogenated amorphous carbon (*a*-C:H) films have optical and electrical properties that vary widely depending on deposition conditions; however, the electrical conduction mechanism, which is dependent on the film structure, has not yet been fully revealed. To understand the relationship between the film structure and electrical conduction mechanism, three types of *a-*C:H films were prepared and their film structures and electrical properties were evaluated. The *sp*^2^/(*sp*^2^ + *sp*^3^) ratios were measured by a near-edge X-ray absorption fine structure technique. From the conductivity–temperature relationship, variable-range hopping (VRH) conduction was shown to be the dominant conduction mechanism at low temperatures, and the electrical conduction mechanism changed at a transition temperature from VRH conduction to thermally activated band conduction. On the basis of structural analyses, a model of the microstructure of *a*-C:H that consists of *sp*^2^ and *sp*^3^-bonded carbon clusters, hydrogen atoms and dangling bonds was built. Furthermore, it is explained how several electrical conduction parameters are affected by the carrier transportation path among the clusters.

## 1. Introduction

Hydrogenated amorphous carbon (*a*-C:H) films are a non-crystalline material [1]. They consist of *sp*^2^ and *sp*^3^-hybridized bonded carbon atoms and hydrogen atoms. The most popular process to synthesize *a*-C:H films is plasma-enhanced chemical vapor deposition (PECVD) [2]. The mechanical and tribological properties of *a*-C:H films depend on their atomic structure [3]. These films, and particularly what is called diamond-like carbon (DLC) films, have outstanding mechanical properties, such as high hardness, low coefficients of friction and excellent corrosion and wear resistance. Fiaschi et al. reported that the hardness of DLC films depends on their *sp*^3^ content and that the hardness affects tribological properties [3]. Many researchers have mentioned that the friction coefficient and specific rate of the films were affected by their hydrogen content [4,5,6]. From these studies, there have been clarifications made of the relationship between the mechanical properties of *a*-C:H film and the *sp*^2^/*sp*^3^ carbon–bonding ratio, and also the relationship between the mechanical properties of *a*-C:H films and the amount of hydrogen. As a result of this clarification, the *a*-C: H film with the optimum mechanical properties for use in a given environment has been applied to the surfaces of mechanical parts. [1,7,8,9]. Another notable feature of *a*-C:H films is that they have a wide range of physical properties resulting from their structural flexibility. The *sp*^2^/*sp*^3^ ratio and hydrogen content can be controlled in the range of 0.3–0.9 and 0.1–0.6, respectively [8,10]. These structural factors depend on the deposition condition [1]. Their electrical and electronic properties also change. The optical band gap and electrical resistivity are in the range of 0.5–4.5 eV [11,12,13], and 10^2^–10^16^ Ω·cm [14], respectively. These films therefore have the potential to be used as semiconductor materials that offer widely variable optical and electrical properties.

The most commonly used amorphous semiconductor material is hydrogenated amorphous silicon (*a*-Si:H). The properties of *a*-Si:H have been studied intensively for electronic applications in industry [15,16,17]. Spear and Le Comber found that *a*-Si:H could be substitutionally doped by boron and phosphorus by adding diborane or phosphine to the source silane gas stream [18]. Hydrogen incorporated in *a*-Si:H terminates the dangling bonds, which causes structural relaxation. As a result, localized states in the band gap significantly decrease and doping is made possible.

Unlike silicon, carbon can form three types of covalent bonds because of its ability to form hybrid orbitals of *sp*, *sp*^2^ and *sp*^3^. In *a*-C:H, there exist mainly *sp*^2^ and *sp*^3^ hybridized carbon simultaneously [8,19]. For *sp*^2^ hybridized carbon, the 2*s* orbital is mixed with two 2*p* orbitals to form three equivalent *sp*^2^ orbitals and one remaining *p* orbital. The overlap of *p* orbitals on adjacent carbon atoms forms π bonds. These π bonds are accompanied by *sp*^2^ hybridization that creates electronic states near the Fermi level and thus determines the electronic properties [20,21,22,23]. The π states are strongly affected by the interatomic distance and atomic coordination. It is therefore expected that the mechanism of electrical conduction in *a*-C:H differs considerably from that of *a*-Si:H. While many *a*-C:H films have been applied in the mechanical field, the scientific reports of electrical and electronic properties of the films are very limited and the relationship between the basic structural factor and these properties is still not clear.

Hydrogenated amorphous carbon began to attract attention as a semiconductor material after *a*-Si:H had already become the most commonly used amorphous semiconductor material [13]. As with *a*-Si:H, *a*-C:H can be synthesized over large areas and grown to have graded properties by controlling deposition conditions during the film growth. Meyerson and Smith found that *a*-C:H could be doped by adding B_2_H_6_ or PH_3_ in a similar manner to *a*-Si:H [24]. In addition, *a*-C:H films can be produced from relatively safer and lower-cost source materials such as hydrocarbon and graphite. Hence, *a*-C:H films are expected to be applied to devices such as solar cells and thin-film transistors (TFT) [13]. Fundamental research focused on the electrical conduction mechanism in *a*-C:H films was conducted by Fabisiak et al. [25]. They showed that the temperature dependence of the electrical conductivity of *a*-C:H films indicate thermally activated band conduction at high temperatures and variable-range hopping (VRH) conduction at low temperatures [25]. The functional relationship for thermally activated band conduction in the extended state is given by the following expression [26]:

(1) $$\sigma_{b}=\sigma_{0}\exp\left(-\frac{E_{a}}{k_{B}T}\right)$$

and the functional relationship for VRH conduction in the localized states near the Fermi level is given by the following expression [27]:

(2) $$\sigma_{h}=\sigma_{00}\exp\left[-{\left(\frac{T_{0}}{T}\right)}^{1/4}\right]$$

where *E*_a_ is the activation energy, *k*_B_ the Boltzmann constant, *T*_0_ the Mott’s characteristic temperature and *T* the absolute temperature.

There are, however, few practical applications of *a*-C:H films in the field of electrical and electronic engineering because their electrical-conduction mechanism has not yet been fully revealed. There has been only a limited number of studies on *a*-C:H films seeking to explain the relationship between their electrical properties and film structure and, in particular, to explain the effect of *sp*^2^/*sp*^3^ ratio and hydrogen content on this relationship. This is thought to be because it is difficult to evaluate the *sp*^2^/*sp*^3^ ratio quantitatively. The effect of the presence of both *sp*^3^ and *sp*^2^ bonding on the properties of the films is still unclear. For electrical and electronic applications using *a*-C:H films to progress, it is important to investigate the relationship between film structure and electrical properties and it is necessary to determine which structures are better for such applications.

In this study, three types of *a*-C:H films were deposited from three different hydrocarbons, and then the relationship between the film structure and conduction characteristics of the films was investigated. To determine the film structure and hydrogen content, the *sp*^2^/(*sp*^2^ + *sp*^3^) ratio and *sp*^2^ cluster size in the films were evaluated. Temperature dependence of electrical conductivity is characterized by several parameters that indirectly reflect the atomic structure: activation energy (*E*_a_), transition temperature of conduction mechanisms (*T*_c_) and Mott’s characteristic temperature (*T*_0_). On the basis of this dependence, a model giving an explanation of how the electrical conduction mechanism will change due to the variation of the structure was proposed.

## 2. Materials and Methods

**Figure 1:**
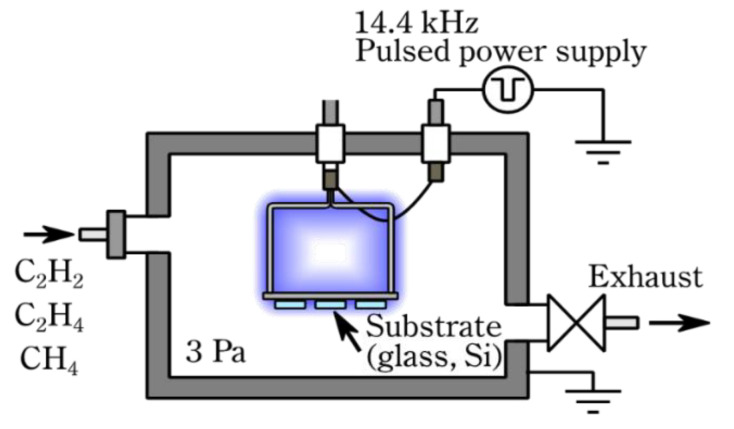
Schematic drawing of a pulsed plasma-enhanced chemical vapor deposition apparatus.

The *a*-C:H films were prepared by pulsed plasma-enhanced chemical vapor deposition (CVD) [28]. We deposited 3 types of *a*-C:H films on 100 *p*-Si substrates (thickness of 625 μm) from acetylene (C_2_H_2_, purity of 98%), ethylene (C_2_H_4_, purity of 99.5%) and methane (CH_4_, purity of 99.999%). The Si substrates were ultrasonically cleaned in distilled water (10 min), then ethanol (10 min) and finally acetone (10 min). This sequence of cleaning was performed twice. The substrates were then placed on a negative electrode in a vacuum chamber, as illustrated in Figure 1. Prior to the deposition, the natural oxidation layer on the Si substrates was removed by argon (Ar, purity of 99.9999%) plasma irradiation for 30 min. Argon gas was introduced at 20 cm^3^/min, and the pressure was maintained at 2 Pa. The applied voltage was −3.5 kV at a frequency of 14.4 kHz. The deposition conditions of the *a*-C:H films are shown in Table 1. Although the temperature during film deposition cannot be measured because the voltage is directly applied to the substrate, it was estimated to be 200 °C or less by using the temperature label for vacuums. The fact that the apparatus could be held in the hand immediately after deposition suggests that the estimated temperature was correct. The composition of the films was determined using glow-discharge optical emission spectroscopy (GDOES, JY-5000RF, HORIBA, Ltd.; Kyoto, Japan) and a near-edge X-ray absorption fine structure (NEXAFS) technique. In GDOES measurements, a calibration curve was used for the evaluation of hydrogen content of the *a*-C:H films. The calibration curve was prepared from the relationship between the hydrogen content and optical-emission intensity of standard samples: pure Si wafer and 2 types of *a*-C:H films, of which hydrogen contents were previously measured by Rutherford backscattering spectrometry (RBS) with elastic recoil detection analysis (ERDA) [28]. The NEXAFS measurements were performed at the beamline 3.2 Ub of the Synchrotron Light Research Institute (SLRI) in Thailand. The NEXAFS spectra were measured in the total electron yield (TEY) mode. The *sp*^2^*/(sp*^2^ + *sp*^3^) ratios of the *a*-C:H films were determined by comparison with the NEXAFS spectrum of highly oriented pyrolytic graphite (HOPG). The *sp*^2^ cluster size of the *a*-C:H films was evaluated using Raman spectroscopy (NRS-4100; JASCO Corp.; Tokyo, Japan, wavelength of 532 nm). In the Raman spectra of *a*-C:H films, the area ratio of the D and G band (*I*_D_/*I*_G_) is inversely proportional to the average grain size of the *sp*^2^ cluster [29]. In this study, multi-Gaussian fitting was used to deconvolute the Raman spectra, and the *sp*^2^ cluster size of the *a*-C:H films was evaluated from the *I*_D_/*I*_G_ ratio.

**Figure 2:**
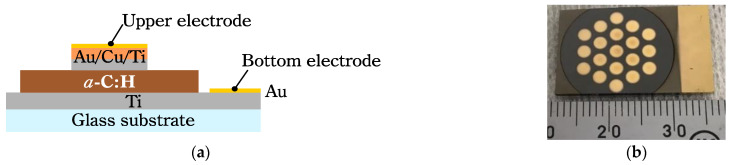
(**a**) Schematic drawing of the cross-section and (**b**) photo of the Ti/*a*-C:H/Ti device for electrical conductivity measurement.

To investigate the temperature dependence of electrical conductivity of the *a*-C:H films, Ti/*a*-C:H/Ti devices were fabricated on glass substrates (S9224, Matsunami Glass Ind., Ltd; Ltd Osaka, Japan). The structure of the device is illustrated in Figure 2. The glass substrate was cut into 15 × 25 × 1.3 mm^3^. These glass substrates were cleaned with pure water, ethanol and acetone in the same way as the Si substrates were cleaned. Upper and bottom Au/Ti electrodes were deposited on the glass using DC magnetron sputtering. A Au (99.99% JEOL Ltd.; Tokyo, Japan) plate with Φ 46.2 mm and Cu and Ti (99.5%, Kojundo Chemical Lab. Co., Ltd.; Sakado, Japan) plates with Φ 100 mm were used as targets. First, the lower titanium electrode was deposited on the glass substrate. Argon was introduced at a rate of 20 cm^3^/min in a chamber and pressure was adjusted to 2 Pa. A DC of 0.4 A and 0.3 kV was applied to the Ti target to generate Ar plasma, and the Ti film was deposited on a glass substrate to form the lower electrode. The deposition duration was 50 min. After that, a stainless steel mask with a hole (Φ 18 mm in diameter) was attached, and an *a*-C: H film with Φ 18 mm was prepared on the Ti electrode under the conditions in Table 1. Then, a stainless steel mask with holes (2 mm diameter) was attached, and Ti and Cu films with Φ 2 mm were sequentially deposited above the *a*-C: H layer for 30 min, under the same conditions as the Ti layer fabrication, to form Cu/Ti electrodes. Finally, Au was deposited as an oxidation protective film on the bottom Ti electrode and the upper Cu/Ti electrode by magnetron sputtering. The sputtering gas was Ar, the voltage was 1.2 kV, the current was 10 mA and the deposition time was 5 min. The sample shown in Figure 2 was obtained. The film thickness was measured by cross-sectional observation using a field emission scanning electron microscope (FE-SEM, JSM-7500F; JEOL Ltd.; Tokyo, Japan). The device was mounted on the cold head of a 4K Gifford-McMahon cryocooler (RDK-101D/CAN-11B; Sumitomo Heavy Industries, Ltd.; Tokyo, Japan) and was connected to an ultra-high resistance meter (R8340A, ADVANTEST Corp.; Tokyo, Japan). The I–V characteristic was measured at RT in the voltage range of 0 ± 0.5 V to confirm the formation of Ohmic contact between *a*-C:H and the electrode system. The temperature dependence of the electrical conductivity of the *a*-C:H films were measured in the range of 30–300 K. The applied voltage was 0.1 V, and the temperature steps were 5 K per step (30–100 K) and 10 K per step (100–300 K). The current measurements were performed after the temperature became stable at the set points.

## 3. Results

**Figure 3:**
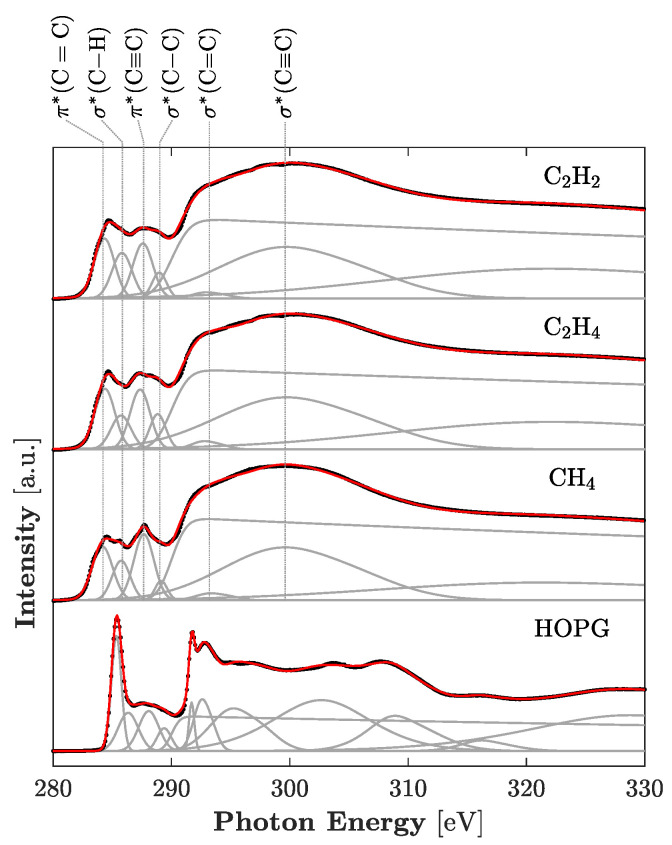
Carbon K-edge NEXAFS spectra of HOPG (standard) and *a*-C:H films deposited from different source materials. The curve fittings of the raw NEXAFS spectra are shown as the gray lines and their sum as red lines.

**Figure 4:**
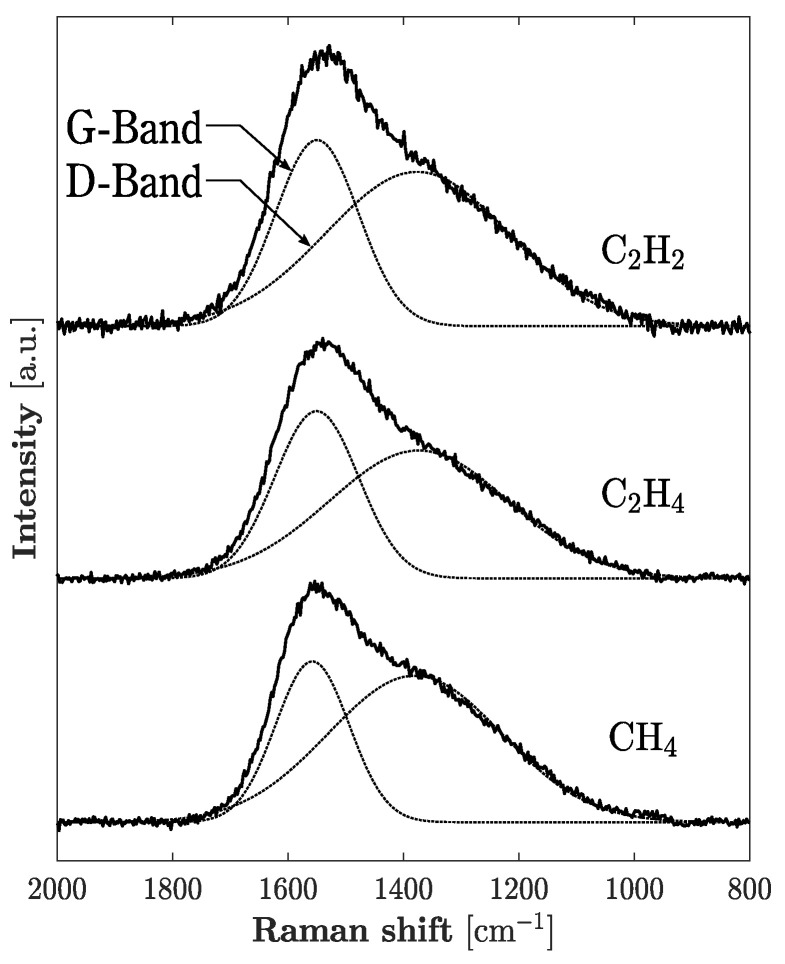
Raman spectra of *a*-C:H films deposited from different source materials. The raw spectra are deconvoluted into 2 Gaussian profiles. The deconvoluted curves are shown as dashed lines.

Figure 3 shows the C *K*-edge NEXAFS spectra of the *a*-C:H films. The analyses of the NEXAFS spectra showed the *sp*^2^/(*sp*^2^ + *sp*^3^) ratio of carbon in the film changed from 68.6% to 69.8% depending on the deposition condition. The spectra were deconvoluted into multiple peaks, and these peaks are assigned to each structure as shown in Figure 3. The presence of pre-edge resonance at a photon energy of 284.6 eV were assigned to the transition from the 1s orbital to the unoccupied π* orbitals that principally originated from the *sp*^2^ site (C=C), and the value included the contribution of the *sp* sites (C≡C) if present [30]. The edge jump from 288.0 to 330.0 eV was related to direct ionization from the 1s orbital [31]. The other peak positions at 286.6, 287.5, 288.8, 293.0 and 303.8 eV were attributed to the σ* (C–H), π* (C≡C), σ* (C–C), σ* (C=C) and σ* (C≡C) states, respectively [32]. The hydrogen content obtained from GDOES analysis changed from 15.3 to 22.9 at.%. The hydrogen content of the films had a tendency to increase as the concentration of hydrogen atoms in the source material increased. Figure 4 shows the Raman spectra of the *a*-C:H films. The value of the *I*_D_/*I*_G_ ratio was calculated after the deconvolution into the D and G band by multi-Gaussian fitting. Structures of the *a*-C:H films are summarized in Table 2. These structures depended on hydrogen content. The *sp*^2^/(*sp*^2^ + *sp*^3^) ratio decreased, and the *I*_D_/*I*_G_ ratio increased with increasing hydrogen content. These results indicate that hydrogen atoms in the films terminated dangling bonds on the surface of *sp*^2^ clusters, and consequently the *sp*^2^ clusters’ size was reduced with increasing hydrogen content.

**Figure 5:**
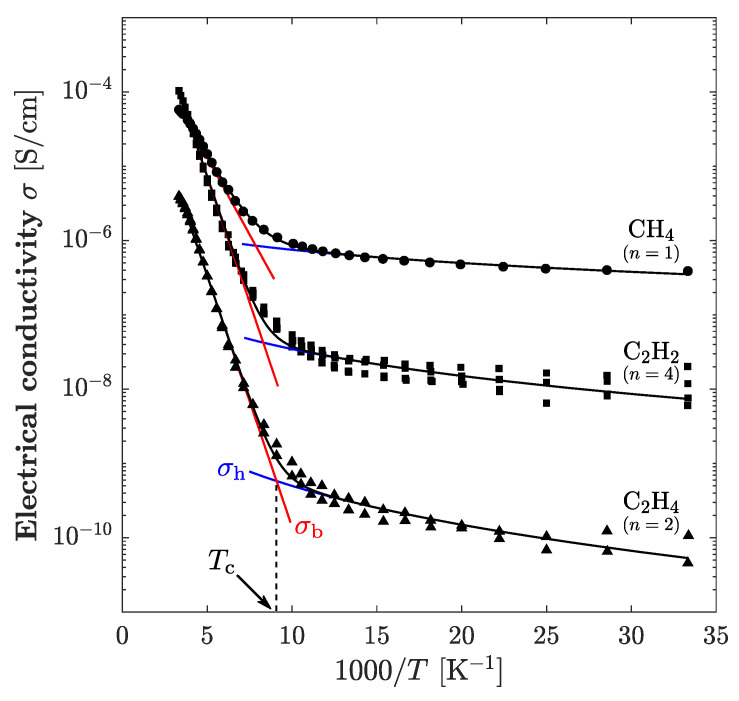
Temperature dependence of electrical conductivity of *a*-C:H films deposited from various source materials. The conductivity of band conduction (σ_b_), VRH conduction (σ_h_) and their sum are represented as blue, red and black lines, respectively. Transition temperature (*T*_c_) is defined as the cross point of σ_b_ and σ_h_ curves. The symbol *n* represents the number of measurements.

The temperature dependences of electrical conductivity were measured for all *a*-C:H films. Figure 5 shows the dependencies in the form of an Arrhenius plot. The temperature dependence of the conductivity changed at a temperature *T*_c_. The temperature dependence above *T*_c_ showed a linear relationship between the log (σ) and the 1/*T*. In contrast, the temperature dependence below *T*_c_ showed a non-linear relationship. As mentioned in the introduction, *a*-C:H films exhibit two different conduction mechanisms. In this study, it was assumed that the conduction at temperatures above *T*_c_ is band conduction and the conduction at temperatures below *T*_c_ is VRH conduction.

The curve fitting of the measured Arrhenius plots was performed using the mixed form of

(3) $$\sigma =\sigma_{0}\exp\left(-\frac{E_{a}}{k_{B}T}\right)+\sigma_{00}\exp\left[-{\left(\frac{T_{0}}{T}\right)}^{1/4}\right]$$

and the electrical conduction parameters of *E*_a_, *T*_0_ and *T*_c_ for each *a*-C:H film were obtained. The values of *E*_a_, *T*_0_, *T*_c_ and the electrical conductivity at 300 K represented as σ_300_ are summarized in Table 3.

## 4. Discussion

**Figure 6:**
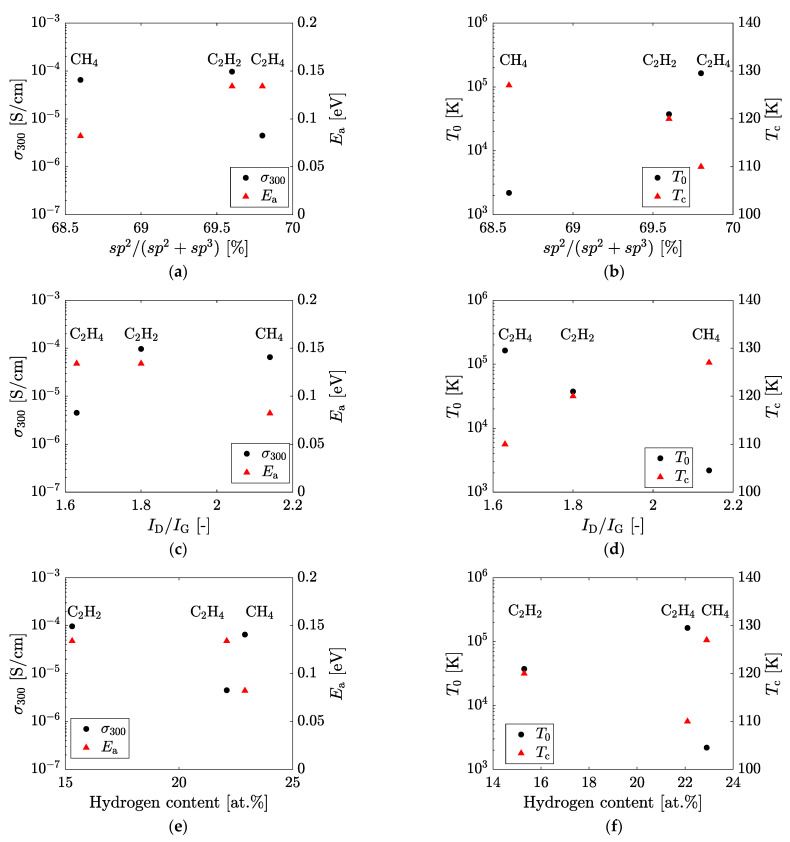
Relationship between the structural factor (horizontal axis) and electrical conduction characteristics (vertical axes). (**a**) *sp*^2^(*sp*^2^ + *sp*^3^) to σ_300_ or *E*_a_, (**b**) *sp*^2^(*sp*^2^ + *sp*^3^) to *T*_0_ or *T*_c_, (**c**) *I*_D_/*I*_G_ ratio to σ_300_ or *E*_a_, (**d**) *I*_D_/*I*_G_ ratio to *T*_0_ or *T*_c_, (**e**) Hydrogen content to σ_300_ or *E*_a_, and (**f**) Hydrogen content to *T*_0_ or *T*_c_.

Figure 6 shows the relationship between the film structure factor (*sp*^2^/(*sp*^2^ + *sp*^3^) ratio, *I*_D_/*I*_G_ ratio and hydrogen content, and electrical conduction characteristics (σ_300_, *E*_a_, *T*_0_ and *T*_c_). As shown in Figure 6a,b, with an increasing *sp*^2^/(*sp*^2^ + *sp*^3^) ratio, *E*_a_ and *T*_0_ show an increasing trend, and σ_300_ and *T*_c_ show a decreasing trend. In Figure 6c,d, with an increasing *I*_D_/*I*_G_ ratio, *E*_a_ and *T*_0_ show a decreasing trend, and σ_300_ and *T*_c_ show an increasing trend. In Figure 6e,d, *E*_a_ tends to decrease as the hydrogen content increases, while σ_300_, *T*_0_, and *T*_c_ do not depend on the hydrogen content. It is necessary to consider that *E*_a_ is related to the band conduction and that *T*_0_ and Tc are mainly related to the VRH conduction. For VRH conduction at low temperatures, Equation (2) can be written as

(4) $$\sigma_{h}=\sigma_{00}\exp\left[-{\left(\frac{T_{0}}{T}\right)}^{1/4}\right]=\sigma_{00}\exp\left[-{\left(\frac{k_{B}T_{0}}{k_{B}T}\right)}^{1/4}\right]$$

In this equation, the dimension of *k*_B_*T*_0_ is energy, and *k*_B_*T*_0_ can be regarded as an energy barrier for carrier transport in VRH conduction. The height and thickness of the energy barrier between the hopping sites simultaneously affects the probability of the occurrence of carrier transport by the phonon-assisted tunneling process. In other words, an increase in *T*_0_ in the low-temperature region corresponds to an increase in the energy barrier and a decrease in the probability of carrier transport occurring, which are both caused by an increase in the distance between the hopping sites.

**Figure 7:**
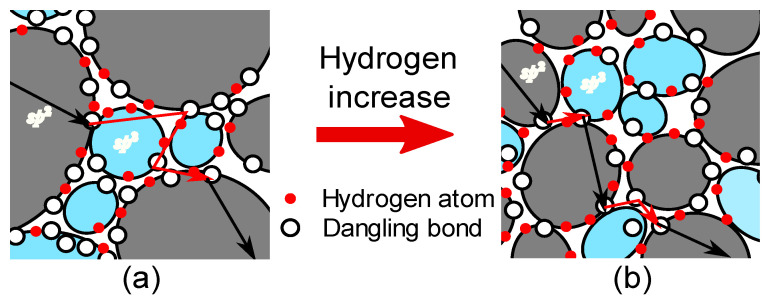
Structure model with conduction pathway. The black and red arrows mean band and VRH conductive pathways, respectively. With increasing of hydrogen atoms from (**a**) to (**b**), the *sp*^2^ cluster size and the *sp*^2^/(*sp*^2^ + *sp*^3^) ratio are reduced, the distance between hopping sites is shorten.

To explain the relationship between film structure and electrical properties, the carrier transport path in the microstructure of *a*-C:H films was considered. On the basis of the results of the structural analyses, the structure model of *a*-C:H film as illustrated in Figure 7 was proposed. This model describes that with increasing hydrogen content the *sp*^2^ clusters’ size is reduced and the *sp*^2^/(*sp*^2^ + *sp*^3^) ratio becomes low. In this model, the carrier moves among the *sp*^2^ and *sp*^3^ clusters by band conduction or VRH conduction. The electrical conductivity of *sp*^2^ clusters in an *a*-C:H film is expected to increase with decreasing temperature, which is what happens with multi-layer graphene [33]. Thermally activated band conduction does not readily occur inside the *sp*^3^ cluster at low temperatures. The mechanism of decreasing electrical conductivity in the low-temperature region is hence assumed to be related to the carrier transport path at the interface between the *sp*^2^ clusters and the *sp*^3^ clusters. The carriers that have passed through the *sp*^2^ clusters are transported through DB on the cluster interface by hopping.

In Figure 6b, the Mott’s characteristic temperature *T*_0_ increases with the increase in the *sp*^2^/(*sp*^2^ + *sp*^3^) ratio. When the *sp*^2^/(*sp*^2^ + *sp*^3^) ratio is small and the *sp*^2^ cluster size is also small, the clusters are close to each other (Figure 7b). The DBs at the cluster interface hence are also close to each other, and thus the energy barrier between the hopping sites is thin. In contrast, when the *sp*^2^/(*sp*^2^ + *sp*^3^) ratio and the *sp*^2^ clutter size become large, the distance between the clusters increases and the hopping distance also increases (Figure 7a), which increases the energy barrier. Therefore, *T*_0_ increases as the *sp*^2^/(*sp*^2^ + *sp*^3^) ratio increases. The transition temperature *T*_c_ tends to decrease as the *sp*^2^/(*sp*^2^ + *sp*^3^) ratio increases, as shown in Figure 6b. The temperature *T*_c_ is at the intersection point of the σ_b_ and σ_h_ curves, and *T*_c_ is hence the temperature at which the main mechanism of carrier transport changes from band conduction to VRH conduction. In Figure 5, the σ_h_ curves shift downward as the *sp*^2^/(*sp*^2^ + *sp*^3^) ratio increases, and consequently, the intersection with σ_b_ curves moves to the right. In this case, the decreasing tendency of *T*_c_ results from the decrease in the pre-exponential factor σ_00_, i.e., the VRH conduction itself. If *T*_0_ is taken into consideration, it suggests that the carrier transport at low temperatures is affected by the size and/or number density of the *sp*^2^ cluster and by the DB distribution at the cluster interface. Hence, it is reasonable to suppose that the increase in the *sp*^2^ cluster size reduces the surface area of the cluster, which results in the reduction of the total number of DBs responsible for VRH conduction. As a result, the *T*_c_ decreases because the σ_00_ decreases as the *sp*^2^/(*sp*^2^ + *sp*^3^) ratio increases. In Figure 6c,d, the changes in the electrical conduction characteristics as the *I*_D_/*I*_G_ ratio increases are opposite to the changes in the electrical conduction characteristics as the *sp*^2^/(*sp*^2^ + *sp*^3^) ratio in Figure 6a,b increases. This is because the *I*_D_/*I*_G_ ratio and *sp*^2^/(*sp*^2^ + *sp*^3^) ratio are closely related to each other due to the *sp*^2^ cluster size but there is a negative correlation between them. In Figure 6e, the activation energy *E*_a_ decreases with increasing hydrogen content. When the hydrogen content is high, the *sp*^2^ cluster is smaller and there are many DBs and hydrogen atoms at the edge of the cluster. In *a*-C:H film, hole transport dominates, and the Fermi level lies nearer the valence band. Additionally, the slope of the valence band tail is sharper than the conduction band tail [19]. As a result of the increase in the number of DBs, the localized states in the band gap become apparent, and the Fermi level approaches the valence band to satisfy charge neutrality. Thus, the *E*_a_ decreases with the increasing termination of the *sp*^2^ clusters by hydrogen atoms. The conductivity at room temperature σ_300_ does not have a clear dependence on the *sp*^2^/(*sp*^2^ + *sp*^3^) ratio or hydrogen content. Since the DB density in the film is large, the electrical conduction mechanism near room temperature is considered to be a superposition of band conduction and VRH conduction. Furthermore, the electrical conductivity of the *a*-C:H film is strongly affected by the state of the *sp*^2^ cluster interface in the film. Thus, σ_300_ may have a complicated tendency.

## 5. Conclusions

The structures of the *a*-C:H films depend on the hydrogen content and electrical conduction properties of the film changes. The electrical conduction mechanism of all of the *a*-C:H films changed, at a transition temperature T_c_, from VRH conduction at low temperatures to band conduction at high temperatures. The changes of *T*_0_ and *T*_c_, which relate to VRH conduction, depend on the size of *sp*^2^ clusters in the films. When the average size of an *sp*^2^ cluster is large, the distance between hopping sites is large and the number density of hopping sites is low. This results in an increase of *T*_0_ and a decrease of T_c_. The changes in *E*_a_, which relates to band conduction, depend on the hydrogen content. When the hydrogen content is high, the hydrogen atoms terminate dangling bonds at the edges of *sp*^2^ clusters and within the *sp*^3^ domain. As a result, the Fermi level becomes closer to the valence band, and *E*_a_ decreases.

## Figures and Tables

**Table 1:** Conditions for plasma CVD of *a*-C:H films.

Source Gas	C_2_H_2_	C_2_H_4_	CH_4_
Base pressure (Pa)	5 × 10^−3^	5 × 10^−3^	5 × 10^−3^
Process pressure (Pa)	3	3	3
Flow rate (cm^3^/min)	20	20	15.5
Applied voltage (kV)	−4.5	−4.0	−4.0
Frequency (kHz)	14.4	14.4	14.4

**Table 2:** Structural characteristics of *a*-C:H films deposited from various source gases.

Source Gas	C_2_H_2_	C_2_H_4_	CH_4_
H content (at.%)	15.3	22.1	22.9
*sp*^2^/(*sp*^2^ + *sp*^3^) (%)	69.6	69.8	68.6
*I*_D_/*I*_G_	1.80	1.63	2.14

**Table 3:** Electrical properties of *a*-C:H films deposited from various source gases.

Source Gas	C_2_H_2_	C_2_H_4_	CH_4_
*σ*_300_ (S/cm)	9.69 × 10^−5^	4.49 × 10^−6^	6.52 × 10^–5^
*E*_a_ (eV)	1.34 × 10^−1^	1.34 × 10^−1^	8.21 × 10^−2^
*T*_0_ (K)	3.74 × 10^4^	1.64 × 10^5^	2.19 × 10^3^
*T*_c_ (K)	120	110	127

## Data Availability

Data sharing not applicable.
